# Structural and Dynamic Insights into the W68L, L85P, and T87A Mutations of *Mycobacterium tuberculosis* Inducing Resistance to Pyrazinamide

**DOI:** 10.3390/ijerph19031615

**Published:** 2022-01-30

**Authors:** Eid A. Alatawi, Fahad M. Alshabrmi

**Affiliations:** 1 Department of Medical Laboratory Technology, Faculty of Applied Medical Sciences, University of Tabuk, Tabuk 71491, Saudi Arabia; eid.alatawi@ut.edu.sa; 2 Department of Medical Laboratories, College of Applied Medical Sciences, Qassim University, Buraydah 51452, Saudi Arabia

**Keywords:** tuberculosis, drug resistance, mutations, molecular docking, MD simulations

## Abstract

Tuberculosis (TB), the most frequent bacterium-mediated infectious disease caused by *Mycobacterium tuberculosis*, has been known to infect humans since ancient times. Although TB is common worldwide, the most recent report by the WHO (World Health Organization) listed the three countries of India, China, and Russia with 27%, 14%, and 8% of the global burden of TB, respectively. It has been reported that resistance to TB drugs, particularly by the *pncA* gene to the pyrazinamide drug due to mutations, significantly affects the effective treatment of TB. Understanding the mechanism of drug resistance using computational methods is of great interest to design effective TB treatment, exploring the structural features with these tools. Thus, keeping in view the importance of these methods, we employed state-of-the-art computational methods to study the mechanism of resistance caused by the W68L, L85P, and T87A mutations recently reported in 2021. We employed a molecular docking approach to predict the binding conformation and studied the dynamic properties of each complex using molecular dynamics simulation approaches. Our analysis revealed that compared to the wildtype, these three mutations altered the binding pattern and reduced the binding affinity. Moreover, the structural dynamic features also showed that these mutations significantly reduced the structural stability and packing, particularly by the W68L and L85P mutations. Moreover, principal component analysis, free energy landscape, and the binding free energy results revealed variation in the protein’s motion and the binding energy. The total binding free energy was for the wildtype −9.61 kcal/mol, W68L −7.57 kcal/mol, L85P −6.99 kcal/mol, and T87A −7.77 kcal/mol. Our findings can help to design a structure-based drug against the MDR (multiple drug-resistant) TB.

## 1. Introduction

Tuberculosis (TB), the most frequent bacterium-mediated infectious disease caused by *Mycobacterium tuberculosis*, has been known to infect humans since ancient times [1]. Although TB is common worldwide, the most recent WHO report listed the three countries of India, China, and Russia, with 27%, 14%, and 8% of the global burden of TB, respectively [2]. Tuberculosis (TB) causes more deaths worldwide than HIV/AIDS, remaining a public health concern. Recently in 2019, the WHO also reported an estimated 10.0 million infections of TB in the worldwide population. Drug resistance remains a major obstacle in the successful treatment of TB. It has been reported that among the reported TB cases, 0.5 million TB patients (78% of those diagnosed) reported multidrug-resistant (MDR) TB [2]. In 2019, 3.3% newly reported patients, and 17.7% of patients being treated were reported to have MDR or (RR) rifampicin-resistanct TB [2].

The process of drug resistance is triggered by a spontaneous mutation in the target genes, including the *panD*, *rpoB* gene, or the *inhA* promoter/*katG* gene. Among the known causes of resistance, resistance to pyrazinamide (PZA) is a prime factor in the serious public health concerns posed by *Mycobacterium tuberculosis* (also known as Mtb). PZA has been shown to have a synergistic impact with rifampin (RIF) and isoniazid (INH) in shortening the time of Mtb therapy from nine to six months. In addition to MDR, XDR-TB strains exhibit fluoroquinolone resistance along with one of the injectable drugs, kanamycin, amikacin, and capreomycin [3]. Pyrazinamide (PZA) is an essential first-line drug that is the most susceptible gene and evolves with new drug-resistant mutations worldwide. It is included in both drug-resistant and drug-susceptible treatment regimens used against *tuberculosis*. PZA shows a better sterilizing effect even against the dormant bacilli in macrophages. This pro-drug (PZA) is converted by the pyrazinamidase enzyme (PZase) to its active form pyrazinoic acid (POA) [4]. POA inhibits the biosynthesis of mycobacterial co-enzyme A by binding to *PanD,* which encodes aspartate decarboxylase enzyme [5]. It has been shown that POA targets another gene called ribosomal protein S1 (rpsA), which plays an essential role in protein translation [6]. For instance, mutations in PZA are primarily correlated with drug resistance in TB [7,8,9]. Advanced DNA sequencing studies reported a number of *pncA* mutations that hinder the development of a genetic-based DST [10,11,12,13] in PZA resistance. Furthermore, *pncA* gene mutations were not detected in phenotypically resistant samples induced by PZA and a suggestive alternative mechanism conferring PZA resistance [14,15]. For instance, the phenomena of drug efflux is a recently discovered approach used by mycrobacterium to cause resistance to multiple drugs, i.e., pyrazinamide, Isoniazid, and Streptomycin. A recently published study investigated the role of the efflux pump in drug resistance by exploring the V219A and S292L mutations in Rv1258c in the presence and absence of efflux pump inhibitors. They discovered that when piperine, an efflux pump inhibitor, was used, then no resistance was observed; however, in the absence of piperine, drug efflux was reported [16]. This conveys the role of the efflux inhibitor in addressing the issue of resistance to anti-TB drugs. Other important properties of the PZA drug are the ability to deplete membrane energy and inhibition of trans-translation [6,17]. This challenges the drug-tolerance ability of persistent bacilli to survive under stressful situations [18]. Similarly, specific gene polymorphisms also correlate with drug-resistance phenotypes by spontaneous exposure to acute levels of antibiotics [19]. Other genetic mutations at the chromosomal level [20] are also highly correlated with drug resistance. Recombinant cloning of these target genes also provides implied evidence in the form of a characterized effect in drug-resistant or drug-susceptible isolates [21] and depicts drug resistance mechanisms. The underlying resistance-conferring mutations also serve as a source to explore specific drug resistance mechanisms, especially in MDR-TB at a genetic level.

Forecasting the effect of mutations on the structure of a protein and its function offers remarkable potential for formulating therapeutic approaches [22,23,24,25,26]. A deep understanding of the biological processes, the relationship between genetic patterns and phenotypic characteristics, and how these systems interact with the host can aid in the development of new and successful treatments [27,28,29,30,31,32]. As proteins are encoded by genes and their biological functionality is also controlled by genes, it is essential to understand that both genomics and proteomics patterns hold significant importance in therapeutics research [33,34]. In this regard, computational algorithms and current machine learning predictions have augmented research against different diseases by providing deep insights into the molecular mechanisms of pathogenesis and diseases progression. Bioinformatics approaches are now being used to investigate the host–pathogen interaction and explore the key features from this biological interaction that can be used to develop treatment options [35,36]. Computational molecular search, formulating novel vaccine candidates, and even in platforms development, computational methods play a noteworthy role [37]. They have increased our understanding of how genomic patterns, phenotypic characteristics, structural modeling, mutation tolerance, and function help to decipher disease mechanisms and devise new treatments [38]. Thus, considering the importance of these methods, we have employed computational modeling and biomolecular simulation methods to study the mechanism of resistance caused by the W68L, L85P, and T87A mutations recently reported in 2021, the new mutations that require scrutiny to understand their features for successful treatment [39]. We employed a molecular docking approach to predict the binding conformation and studied the dynamic properties of each complex using molecular dynamics simulation approaches. Our findings would help to design structure-based drugs against the MDR TB.

## 2. Material and Methods

### 2.1. Retrieval of PZase and PZA

The 3D structure of PZase from *Mtb* in an apo form containing Fe ion only was retrieved from RCSB using the PDB ID 3PL1 [40]. Structural assessment for any breaks and missing residues was performed using Chimera v1.15 (University of California, California, CA, USA). Any water molecule in the structure was removed, and the structure was further subjected to correction of protonation states. The prepared wild type structure was then used for mutant modelling using Chimera software (University of California, California, CA, USA) [41]. Using conjugate gradients (1000 steps) and steepest descent methods (1000 steps), the mutant structures were minimized and saved for docking. The SDF file of PZA (pyrazinamide) was obtained from the PubChem database (accession ID: CID1046).

### 2.2. Molecular Docking

Before molecular docking, we used Open Babel software v 8 (University College Cork, Co. Cork, Ireland) to prepare and minimize drug molecules by employing the Universal Force Field (UFF). Then for optimization, the conjugate gradient algorithm was employed. The prepared structure of PZA was docked against PZase by defining the active site residues for grid generation using the AutoDock Software (The Scripps Research Institute, La Jolla, CA, USA) [42]. Exhaustiveness was set high (64) for better accuracy. Finally, the resulting top-scoring conformations were used for simulation purposes using AMBER20 software (University of California, California, CA, USA).

### 2.3. Molecular Dynamics Simulation

Investigation of dynamic features of the wildtype and mutant complexes were explored through molecular dynamics simulation by employing the AMBER20 simulation tool (University of California, California, CA, USA) [43]. For protein parameterization FF19SB, the GAFF (general amber force field) was used for drug topologies generation. The Fe ion parameters were also generated and “optimal” point charge (OPC) waterbox (10 Å) and Na^+^ ions were added to solvate to neutralize each system. A total of 7256 water molecules and 15 sodium ions were added to the 1373 total atoms. Iterative cycles were completed, each step had 6000 cycles employing the steepest descent algorithm and then the conjugate gradient algorithm for another round of 3000 cycles. Each complex was heated to 300 K, with equilibration at constant pressure 1 atm and weak restraint, while in the second round of equilibration, no restraint was added. Lastly, a 100 ns production run was completed. The temperature was controlled with a Langevin thermostat [36]. Long-range electrostatic interactions were treated with a particle mesh Ewald algorithm with a cutoff distance of 10.0 Å [44]. Finally, a SHAKE algorithm was recruited to treat a covalent bond [45]. The GPU (graphical processing unit)-reinforced simulation for each system was completed, while CPTRAJ and CPPTRAJ modules were used to investigate the trajectories [46]. The simulations were performed at the College of Applied Medical Sciences, Qassim University using an HP Z800 Xeon workstation (Hewlett-Packard, California, CA, USA) with 32 GB RAM and Asus extreme gaming 1080 Ti GPU. The simulation of each complex was completed in 3 days and post-simulation analyses were performed in 1 day.

### 2.4. Principal Component Analysis

Using the CPPTRAJ package (University of California, California, CA, USA), the PCA of each complex was performed to map the high amplitude oscillating motions [47]. The covariance matrix was computed and diagonalized to estimate the eigenvectors in each complex and their respective eigenvalues. The whole trajectory of each complex was used to capture high amplitude fluctuations. PC1 and PC2 were computed and plotted against each other to demonstrate the motions.

### 2.5. Free Energy Landscape (FEL)

The free energy landscape (FEL) approach was used to extract the lowest energy state stable conformation shown by the contour. The intermediate conformations are represented by the separating boundaries between the subspaces [48]. The *g_sham* module integrated in Gromacs was employed to calculate the FEL. The first two PCs were then used to calculate the FEL based on the following equation:

$$∆G\left(\mathrm{PC}1,\;\mathrm{PC}2\right)=K_{B\;}\ln P\left(\mathrm{PC}1,\;\mathrm{PC}2\right)$$

### 2.6. Binding Affinity Calculations

The total binding energy of the wildtype and W68L, L85P, and T87A mutant complexes with the PZA drug was calculated through MMPBSA.py script (University of Florida, Gainesville, FL, USA) [49,50,51,52,53,54]. For free energy computation from each trajectory, 5000 structures were subjected to calculation of TBE based on the following equation:

$$“∆G_{\mathrm{bind}}=∆G_{\mathrm{complex}}-\left[∆G_{\mathrm{receptor}}-∆G_{\mathrm{ligand}}\right]”$$

The subsequent equation was used to compute the value of each component:

$$“G=G_{bond}+G_{ele}+G_{vdw}+G_{pol}+G_{npol}”$$

## 3. Results and Discussion

**Figure 1:**
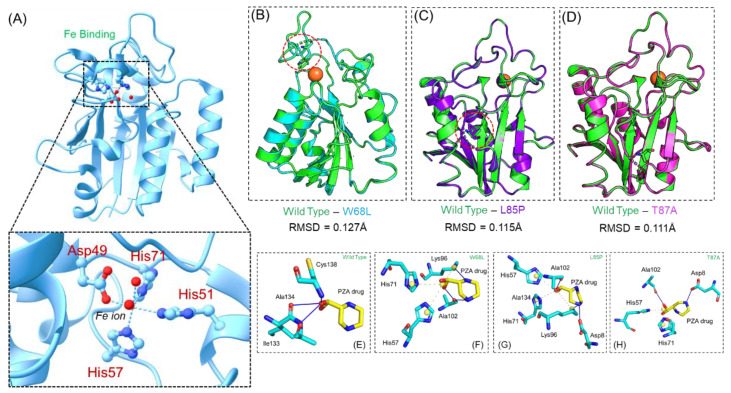
Three-dimensional structure and binding of PZA to the wildtype and W68L, L85P, and T87A mutants. (**A**) The wild type structure of PZase with Fe^2+^ ion. (**B**–**D**) The superimposed structures of the wildtype and three mutants. It also shows the mutations as sticks. (**E**) The binding of the wildtype PZase and PZA, while (**F**–**H**) represent the binding of PZA to W68L, L85P, and T87A mutants. (RMSD = root mean square deviation).

The apo structure of PZase using PDB ID 3PL1 was retrieved from PDB and the mutations, i.e., W68L, L85P, and T87A were generated using the Chimera rotamers tool. Figure 1A represents the 3D structure of wildtype PZase and Fe^2+^ binding. The modeled 3D structures of the mutants are shown in Figure 1B–D, where the mutations are encircled in red. Prior to docking conformation predictions, the structures were minimized and any bad contact was addressed. Superimposition of the wildtype on the three mutants revealed variations in the structures. A superimposition of the wildtype on W68L revealed an RMSD (root mean square deviation) of 0.127 Å, the wildtype and L85A demonstrated an RMSD of 0.115 Å, while the T87A reported an RMSD of 0.111 Å respectively. The docking of the PZA drug was against the active site residues His71, Lys96, Ala134, His137, and Cys138 residues. Two essential residues, i.e., His137 and Cys138 are involved in direct hydrogen bonding interactions with PZase. The docking results revealed that PZA binds to the wildtype more robustly than the three mutants. The docking score for the wildtype was −6.07 kcal/mol; for W68L, it was −5.65 kcal/mol; for L85P, it was −5.73 kcal/mol; while for T87A the docking score was −5.72 kcal/mol. Figure 1E–H shows the variations in the binding pattern of PZA to the wildtype and the three mutants. The wildtype established three essential interactions with ile133, Ala134, and Cys138, which have also previously been reported to interact and produce inhibitory properties. On the other hand, the W68P-PZA complex has three hydrogen bonds (Lys96 and Ala102) and three salt bridges (His57 and His71). This shows that the binding of PZA was altered by the leucine substitution at position 68 and lost the essential interactions with Cys138. Moreover, the binding of L85P also revealed significant variations. With three salt bridges (His57, His71, and Ala134) and three hydrogen bonds (Asp8, Lys96, and Ala102) the docking score of L85P remained relatively higher than W68L. Many salt bridges were established by this mutant; however, due to a lower energy contribution the binding was altered. Moreover, the T87A complexes also established several interactions including hydrogen bonds by Asp8 and Ala102, which were actively involved in the hydrogen bonding. The residues His57, His71, and Ala102 also established salt bridges in this complex. Hence, the docking and binding analysis confirmed that these particular substitutions W68L, L85P, and T87A affected the binding of the PZA. Similar findings of altered binding networks by other mutations by different studies have also been reported previously [55,56,57]. Moreover, we also calculated the binding cavity volume and surface area of all the structures including the wildtype and mutants. The results revealed that the wildtype had a volume of 141.229, the W68L mutant had 136.730, the L85P mutant reported 135.669, while the T87A mutant had 138.945. The findings are consistent with the previous reports, which also confirmed a reduction in the binding cavity volume for the drug-resistant mutations in PZA [56,58]. The top scoring conformations shown in Figure 1 were further used to check the dynamic features altered by these mutations and to demonstrate the binding variation using MM/GBSA.

### 3.1. Stability Evaluation of the Wildtype and Mutant Complexes

**Figure 2:**
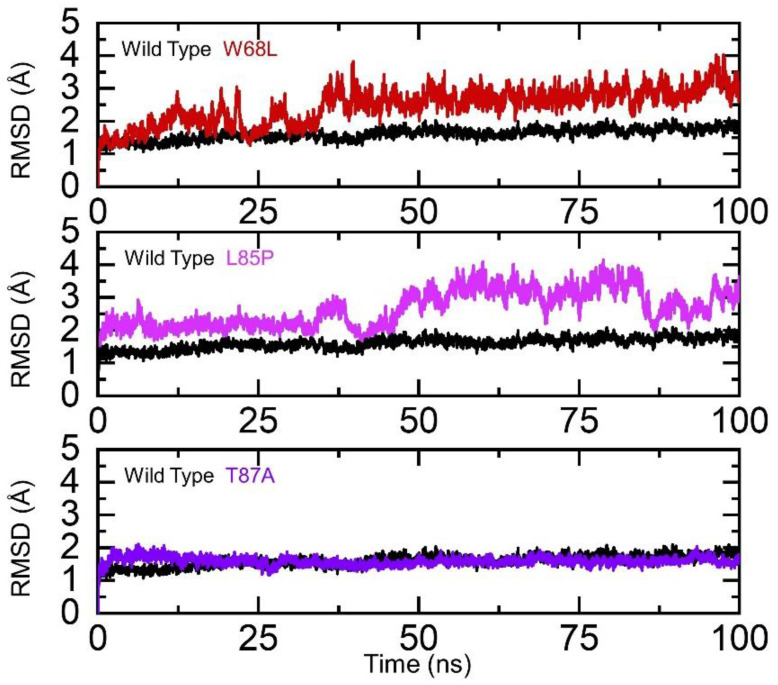
Stability analysis of the wildtype and W68L, L85P, and T87A mutants. The wildtype is shown as black, W68L (red), L85P (magenta), and T87A (purple).

**Figure 3:**
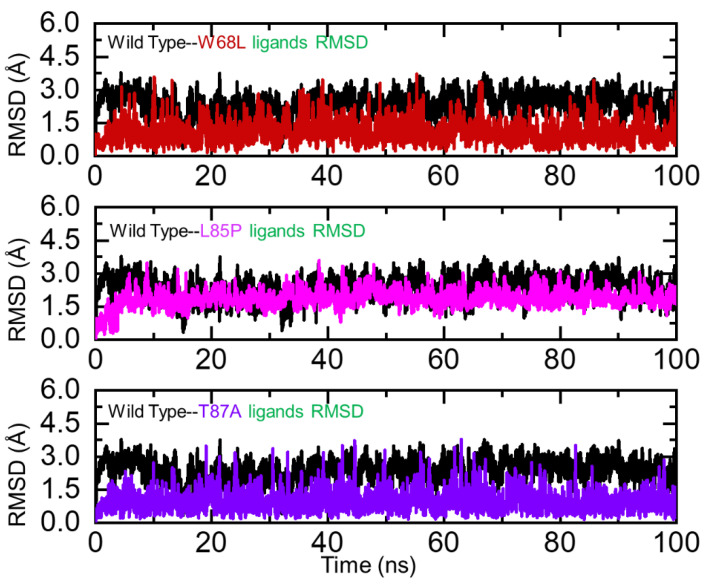
Ligand stability analysis in the binding cavity of the wildtype and W68L, L85P, and T87A mutants. The wildtype is shown as black, W68L (red), L85P (magenta), and T87A (purple).

Assessment of structural stability in a dynamic environment always reveals important information regarding the binding pattern and impact of different mutations. To demonstrate the impact of the aforementioned mutations on the binding complexes, we also calculated the RMSD for each complex. Our analysis revealed that the wildtype complex remained more stable than the mutant complexes. It can be seen that the RMSD of the wildtype exhibited a rigid behavior and no significant deviation was experienced by the system. The complex demonstrated stable dynamics with an average RMSD of 1.2 Å. Comparative assessment of wildtype and W68L revealed significant differences in the stability of the mutant complex. The W68L complex faced significant structural perturbation during the first 35 ns and, then, although the RMSD increased gradually, no significant deviation was experienced. The average RMSD for W68L was reported to be 2.5 Å. Moreover, the L85P complex initially demonstrated rigid and stable dynamics until 40 ns but then the RMSD deviated by a larger proportion and significant convergence was experienced by this complex. The average RMSD for L85P was reported to be 2.4 Å. On the other hand, the T87A and wildtype RMSD remained comparable. The complex did not converge, and the average RMSD was reported to be 1.1 Å. The findings are consistent with previous studies, which also reported that drug resistant mutations destabilized the structures in a dynamic environment, thus implying that these mutations produce destabilizing effects and, consequently, pose resistance to PZA [55,56,57]. The RMSD graphs of the wildtype and mutant complexes are shown in Figure 2. We also calculated the RMSDs of the ligands only, which confirmed the stability of the ligands during the simulation. Binding and unbinding of the ligands during the simulation resulted in an increase or decrease in the RMSD, particularly in the mutant complexes, as can be seen at different time intervals. The RMSDs of the ligands are shown in Figure 3. Moreover, the secondary structural elements were also calculated, which revealed further variations induced by the mutations. In the case of the wildtype, 32.43% of the residues were alpha-helix, 19.46% extended strand, and 8.11% beta sheets, while 40% were random coiled. In the case of the W68L mutant, 34.59% of the residues were alpha-helix, 17.30% extended strand, and 7.57% beta sheets, while 40.54% were random coiled. Consequently, the percentage of beta sheets was reduced, which is directly associated with protein stability. In the case of L85P and T87A, 35.14% and 37.84% of the residues were alpha-helix, respectively, 17.30% and 18.38% were extended strand, and 7.57% and 17.30% were beta sheets, while 38.92% and 34.93% were random coiled. It can be seen that the number of beta sheets was increased in the T87A complex, which in turn increased the stability, as can be seen in the RMSD plot, where the RMSD graph is comparable with the wildtype.

### 3.2. Determination of Structural Compactness as Rg (Radius of Gyration)

**Figure 4:**
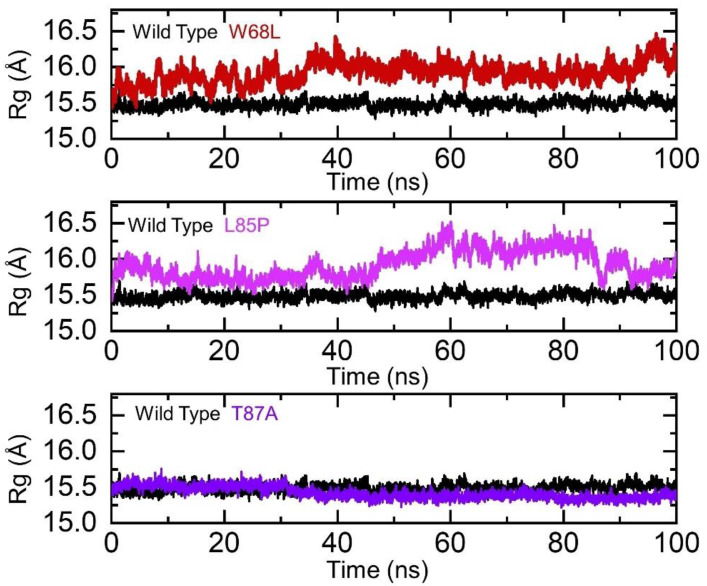
Structural packing of the wildtype and W68L, L85P, and T87A mutants. The wildtype structure is shown as black, W68L (red), L85P (magenta), and T87A (purple).

Determination of structural compactness in a dynamic environment demonstrates important binding and unbinding events and the impact of different mutations [59,60]. To demonstrate the binding and unbinding events, we calculated the radius of gyration (Rg) for each complex as a function of time. The Rg results of the wildtype and mutants are strongly in uniformity with the RMSD results. The Rg(s) of the wildtype and T87A demonstrated similar structural compactness. The average Rg for the wildtype and T87A was reported to be 15.52 Å and 15.50 Å, respectively. Moreover, the L85P and W68L experienced significant variations and perturbations in the Rg during the simulation. The Rg of the W68L gradually increased over the simulation time and reached 16.0 Å. During the first 35 ns, the average Rg value was 15.8 Å and then increased during the last 65 ns up to 16.1 Å. The increment in structural compactness after 90 ns was due to the reduction in the motion of the lid and flap region of the protein structure, which stabilized at that particular time point, hence experiencing reduction in the structural compactness. On the other hand, the Rg pattern of T87A also revealed a consistent graph as did the RMSD. Initially, the structure remained well packed until 45 ns, with a minor deviation, and then, a significant deviation until 100 ns was reported. The average Rg for the T87A complex was reported to be 15.8 Å. The Rg graphs of the wildtype and the mutant complexes are shown in Figure 4.

### 3.3. Residual Flexibility (RMSF) Estimation

**Figure 5:**
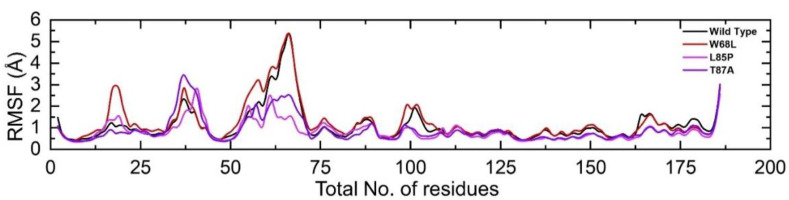
Residual flexibility of the wildtype and W68L, L85P, and T87A mutants. The wildtype structure is shown as black, W68L (red), L85P (magenta), and T87A (purple).

Estimation of residual flexibility is an important parameter to reveal the conformational flexibility and strength conferred by a particular residue in the binding [61]. Herein, to calculate the residual flexibility we used the root mean square fluctuation (RMSF) approach. The results, as shown in Figure 4, revealed that the flexibility of the wildtype and mutant complexes remained comparable; however, variations in different regions particularly 15−25, 30–45, 55–75, and 90–105 demonstrated higher fluctuation in the wildtype and W68L, specifically. The other variants demonstrated higher fluctuation between 30 and 45 aa. The mutation W68L in the loop region resulted in higher fluctuation of the loop, thus rendering higher fluctuation in this particular mutation. In conclusion, the mutations affected the residual flexibility of the protein structure differently and caused functional variations. The RMSF graph of all the complexes is shown in Figure 5.

### 3.4. Hydrogen Bonding Analysis

**Figure 6:**
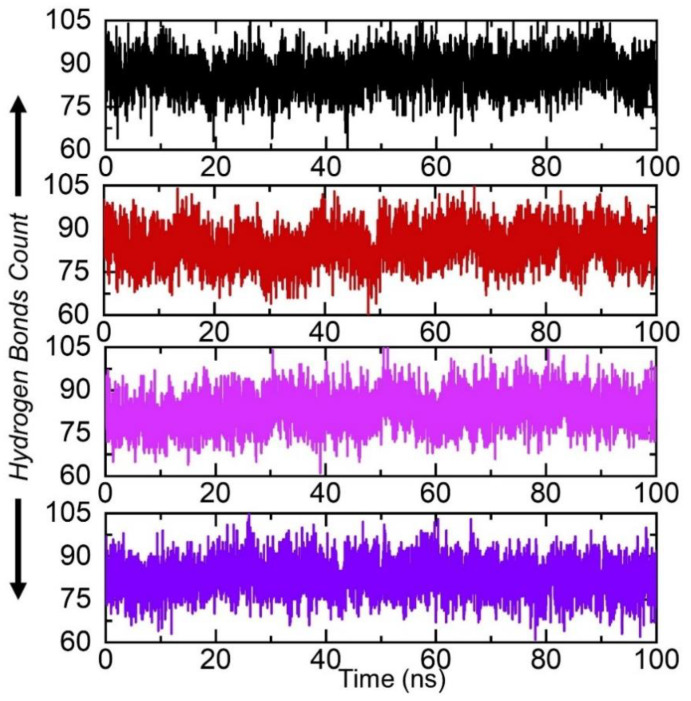
Hydrogen bonding analysis of the wildtype and W68L, L85P, and T87A mutants. The wild type structure is shown as black, W68L (red), L85P (magenta), and T87A (purple).

Analysis of the hydrogen bonds of the protein–ligand trajectories is essential to understand the binding variations produced by epigenetics or mutations [62]. To quantify the total number of hydrogen bonds and reveal the average, we subjected the simulation trajectories to hydrogen bonding calculations as a function of time. Our results revealed that the wildtype and W68L, L85P, and T87A had a different total number of hydrogen bonds, as shown in Figure 5. In each complex, the average hydrogen bonds were estimated to be 93 (wildtype), 90 (W68L), 82 (L85P), and 88 in the T87A mutant. This shows that the mutations destabilized the binding and, thus, caused resistance to PZA. Similar findings were also reported by previous studies, thus justifying our results [55,56,57]. The hydrogen bonds graph of all the complexes is shown in Figure 6.

### 3.5. Principle Component Analysis (PCA) and Free Energy Landscape (FEL)

**Figure 7:**
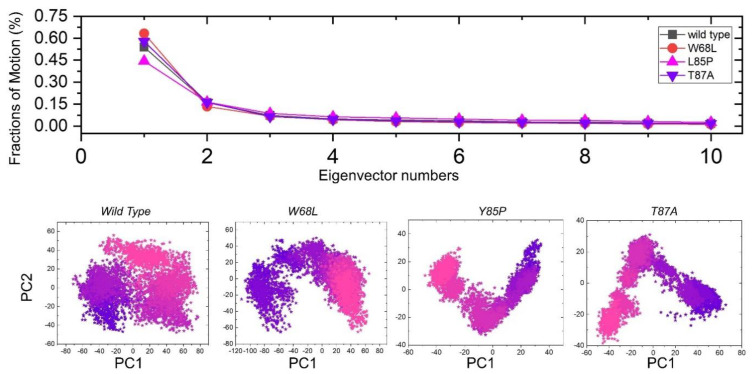
Principal component analysis (PCA) of all the complexes, i.e., wildtype, W68L, L85P, and T87A. The first PC1 and second PC2 from the PCA of the backbone carbon were used.

To identify the collective motions of the wildtype and mutant complexes, we explored the dynamic behavior using PCA. It is a statistical method for dimensionality reduction without compromising vital information [63]. Plentiful fluctuations were revealed by the first three eigenvectors, while localized fluctuations were demonstrated by the other eigenvectors in each complex, as shown in Figure 6. In the case of the wildtype-PZA complex, the first 3 eigenvectors were 53%, the W68L-PZA complex demonstrated 63%, L85P displayed 44%, and T87A 57% of the overall observed motion. This behavior may explain the structural rearrangement due to the mutations; thus, it may empirically be proposed that the PZA interaction may destabilize the protein by increasing the dynamics of the active regions to a higher level. The current findings also support the previous reports with higher magnitude of motion in the PZA complexes and directions identical to the wildtype [55,56,57,58]. Moreover, we also explored the principal components, i.e., PC1 and PC2 (principle components) of the simulation trajectories to project each frame in a phase space (Figure 7). The interpretation of the pink to purple displays the conformational transformations during the simulation. These conformational states were found closer to each other in the three mutants and were considered as an energetically unstable conformational state by attaining an unstable conformational state purple color.

### 3.6. Free Energy Landscape (FEL) Analysis

**Figure 8:**
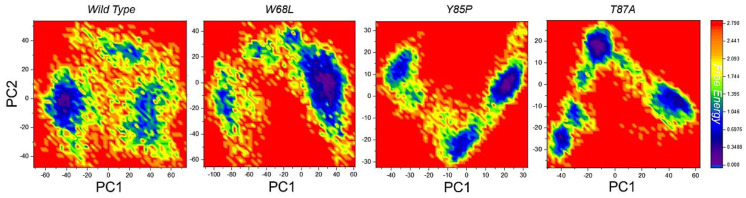
Free energy landscape (FEL) of all the complexes, i.e., wild type, W68L, L85P and T87A. The first PC1 and second PC2 from the PCA of the backbone carbon were used.

In addition, a free energy landscape (FEL) was created to link structural characteristics to thermodynamic attributes. FEL was used to map the minimum energy conformation of the complexes within the examined time scale, and then to connect the structural shifts between these minima based on the likelihood of provided data points of the MD trajectories. Figure 8 presents the FEL of the wildtype, W68L, L85P, and T87A where all reached only one energy minima, thus explaining global conformational variations adjusted by the mutant complexes in response to mutations.

### 3.7. Free Energy Calculations

Using an MM/GBSA (molecular mechanics generalized Born and surface area continuum solvation) approach to re-evaluate the TBE of a protein and ligand is a common approach [1,2,64]. The TBE (total binding energy) was calculated for wildtype, W68L, L85P, and T87A-PZA complexes. The ΔG_bind_ of wild type, W68L, L85P, and T87A-PZA complexes was reported to be −9.61 kcal/mol, −7.57 kcal/mol, −6.99 kcal/mol, and −7.77 kcal/mol, respectively (Table 1). When comparing the overall energies of mutants to the WT, it is clear that these mutations reduce the PZA’s binding strength. In comparison to the WT, the contributions of vdW (Van der Waal), Electrostatic, and PS (polar solvation) energies to the binding energies of the mutants were substantially lower. It was discovered that the mutant proteins have a low affinity for PZA. The configuration of the active site residues in direct contact with the PZA is affected by mutations that are not engaged in direct interaction with the PZA. The current findings strongly corroborate the previous findings where reduction in the binding free energy was reported to be induced by different mutations [55,56,57,58].

## 4. Conclusions

Forecasting the effect of mutations on the structure of a protein’s function and binding offers prodigious potential for formulating therapeutic approaches. A thorough knowledge of biological processes, the relationship between genetic patterns and phenotypic characteristics, and how these systems interact with the host can aid in the development of new and successful treatments. As proteins and their biological functionality are controlled by genes, it is essential to understand that both genomics and proteomics patterns hold significant importance in therapeutics research. Thus, keeping in view the importance of these methods, we have employed advanced computational methods to study the mechanism of resistance caused by the W68L, L85P, and T87A mutations recently reported in 2021. We employed a molecular docking approach to predict the binding conformation and studied the dynamic properties of each complex using molecular dynamics simulation approaches. Our analysis revealed that compared to the wildtype, these three mutations altered the binding pattern and reduced the binding affinity. Moreover, principal component analysis, free energy landscape, and the binding free energy results revealed variation in the protein’s motion and the binding energy. The total binding free energy for the wildtype was −9.61 kcal/mol, W68L −7.57 kcal/mol, L85P −6.99 kcal/mol, and T87A −7.77 kcal/mol. Our findings could help to design structure-based drugs against the MDR TB. Using these structural features, drugs which can induce structural stability and particularly the Fe ion stability could effectively overcome the drug resistance problem. This study offers a profound understanding on the mechanism of resistance caused by the reported mutations in *tuberculosis*.

## Figures and Tables

**Table 1:** Binding free energy results calculated as MM/GBSA of the wildtype and W68L, L85P, and T87A systems represented in kcal/mol.

Complex	vdW	Elec	ΔPS	SASA	TBE
Wild Type	−10.01	−20.11	21.52	−1.01	−9.61
W68L	−8.22	−18.52	20.15	−0.98	−7.57
L85P	−6.34	−19.71	22.02	−2.96	−6.99
T87A	−9.36	−16.35	19.96	−2.02	−7.77

## Data Availability

The data presented in this study are available within the article.
